# Prenatal exposure to fine particles and polycyclic aromatic hydrocarbons and birth outcomes: a two-pollutant approach

**DOI:** 10.1007/s00420-016-1192-9

**Published:** 2017-02-07

**Authors:** W. A. Jedrychowski, Renata Majewska, J. D. Spengler, David Camann, E. L. Roen, F. P. Perera

**Affiliations:** 1 Former Department of Epidemiology, Chair of Epidemiology and Preventive Medicine, Jagiellonian University Medical College, Krakow, Poland; 2 Department of Epidemiology, Chair of Epidemiology and Preventive Medicine, Jagiellonian University Medical College, 7, Kopernika Street, Krakow, Poland; 3 Department of Environmental Health, School of Public Health, Harvard University, Boston, MA USA; 4 Department of Analytical and Environmental Chemistry, Southwest Research Institute, San Antonio, TX USA; 5 Columbia Center for Children’s Environmental Health, Mailman School of Public Health, Columbia University, New York, NY USA

**Keywords:** Birth outcomes, Prenatal exposure, PM2.5, PAH, Krakow cohort

## Abstract

**Background:**

Previous epidemiologic studies have considered the effects of individual air pollutants on birth outcomes, whereas a multiple-pollutant approach is more relevant to public health policy.

**Objectives:**

The present study compared the observed effect sizes of prenatal fine particulate matter (PM_2.5_) and polycyclic aromatic hydrocarbons (PAH) (a component of PM_2.5_) exposures on birth outcome deficits, assessed by the single vs. two-pollutant approaches.

**Methods:**

The study sample included 455 term infants born in Krakow to non-smoking mothers, among whom personal exposures to PM_2.5_ and PAH were monitored in the second trimester of pregnancy. The exposure effect estimates (unstandardized and standardized regression coefficients) on birth outcomes were determined using multivariable linear regression models, accounting for relevant covariates.

**Results:**

In the single-pollutant approach, each pollutant was inversely associated with all birth outcomes. The effect size of prenatal PAH exposure on birth weight and length was twice that of PM_2.5,_ in terms of standardized coefficients. In the two-pollutant approach, the negative effect of PM_2.5_ on birth weight and length, adjusted for PAH exposure, lost its significance. The standardized effect of PAH on birth weight was 10-fold stronger (*β* = −0.20, *p* = 0.004) than that estimated for PM_2.5_ (*β* = −0.02, *p* = 0.757).

**Conclusion:**

The results provide evidence that PAH had a greater impact on several measures of fetal development, especially birth weight, than PM_2.5_. Though in the single-pollutant models PM_2.5_ had a significant impact on birth outcomes, this effect appears to be mediated by PAH.

**Electronic supplementary material:**

The online version of this article (doi:10.1007/s00420-016-1192-9) contains supplementary material, which is available to authorized users.

## Introduction

The number of epidemiologic studies on the adverse impact of air pollutants on population health has grown rapidly over the last decades; and they have provided strong evidence that both short and long-term exposures to airborne pollutants are associated with greater mortality and morbidity risks in adults and children (Dockery et al. 1993; Glinianaia et al. 2004; Katsouyanni et al. 1997; Laden et al. 2000; Pope 2000; Sarnat et al. 2006). Reproductive epidemiology studies on the role of maternal exposure to particulate matter (PM) and other air pollutants in adverse birth outcomes have produced convincing data on the harmful impact of these pollutants on fetal development (Bobak and Leon 1999; Dejmek et al. 2000; Glinianaia et al. 2008; Gouveia et al. 2004; Leem et al. 2006; Parker et al. 2005; Pereira et al. 1998; Ritz et al. 2002; Vrijheid et al. 2011; Wang et al. 1997; Woodruff et al. 1997). The developing fetus is known to be especially susceptible to adverse toxicologic consequences of many environmental chemicals because of elevated cell proliferation rates, reduced capability to detoxify toxic chemicals, diminished immune-response capacity, and physiologic immaturity (Barr et al. 2007; Makri et al. 2004). For example, studies have shown that the fetus, infants and young children are highly vulnerable to the toxic effects of active smoking and environmental tobacco smoke (ETS) (Jedrychowski and Flak 1996; Jedrychowski et al. 1977; Lowe 1959; Martin and Bracken 1986; Ogawa et al. 1991; Rubin et al. 1986; Windham et al. 1999), polycyclic aromatic hydrocarbons (PAH) (Choi et al. 2006; Dejmek et al. 2000; Perera et al. 1998, 2003, 2004), and PM (Bobak 2000; Dejmek et al. 1999; Jedrychowski et al. 2004; Ritz et al. 2000; Rogers et al. 2000). The second trimester of pregnancy marks the halfway period of pregnancy when the fetus starts to grow very quickly and undergoes its most important period of growth. From then, the fully developed placenta provides all the fetus’ needs until birth. This may create favorable conditions for toxicants absorbed by mother to cross the placenta and put the fetus at risk.

Although many studies have confirmed the association between maternal exposure to PM or PAH compounds and adverse birth outcomes, preterm birth, congenital anomalies and growth restriction, some authors have pointed to inconsistency in the methods used in the studies, mainly regarding exposure misclassification and confounding variables (Woodruff et al. 2009). For example, Glinianaia et al. in their systematic review (Glinianaia et al. 2004) identified twelve relevant publications published between 1966 and 2001 in English, which included original data on birth weight, gestational age at delivery, or stillbirth related to exposure to PAH, total suspended particles (TSP), PM_10_, and PM_2.5_. The authors found “little consistency in the evidence linking particulate air pollution and fetal outcomes”. Many studies had methodological weaknesses in their design and adjustment for confounders. Even in well-designed studies, the reported magnitudes of the effects were small and inconsistently associated with exposure at specific stages of pregnancy.

Among hazardous urban air toxics, PAH are of global concern due to their multiple effects on human health. Concern is greatest for PAH bound to PM_2.5_ and particularly the ultrafine fraction (UFF) of PM (Briggs et al. 2008; Hesterberg et al. 2006). About 80% of PM in the urban environment is in the fine (PM_2.5_) and ultrafine (PM_0.1_) mode (Morawska et al. 1998).

To date, few studies have used individual (personal) estimation of fetal exposure to air pollutants; most have relied on proxy exposure data to certain pollutants, which were routinely monitored within a given residential area and often based only on one or two monitoring stations. Moreover, in many studies the main pollutant exposure was limited to PM, which irrespective of its size range, is in fact a multi-pollutant agent containing a varying mixture of chemicals. As TSP, PM_10_, and PM_2.5_ represent somewhat different sources, with crustal materials falling generally in the larger size and combustion particles falling in the smaller range (<2.5 µm), this creates problems in interpreting results for public health policy in causal terms.

Up to now, worldwide public health policies regarding air pollution hazards have generally been directed at controlling single pollutant exposures, although the polluted air we breathe is a complex mixture of pollutants in solid and gaseous states. The single-pollutant studies are relatively straightforward to conduct, and their results are easy to understand. However, this approach fails to consider the total population health responses resulting from the simultaneous exposure of two or more pollutants and does not account for the relationship between given pollutants in the context of specific health risks.

The aims of the study were: to assess which of the personally monitored prenatal pollutants (PM_2.5_ and PAH) is the better predictor of birth outcomes and to test the hypothesis that prenatal PAH exposure is the main etiological agent responsible for adverse birth outcomes. In the present analysis, data from our prospective birth cohort study in Krakow (2000–2004) were analyzed (Jedrychowski et al. 2003).

## Materials and methods

The prospective cohort study design and detailed selection of the population are previously described (Jedrychowski et al. 2003). Briefly, this study is part of an ongoing comparative longitudinal investigation of the health impacts of prenatal exposure to outdoor/indoor air pollution on infants and children in New York City and Krakow. The Jagiellonian University and the Columbia University Medical Center ethics committees approved the study. Data from 505 women who delivered between 34 and 42 weeks of gestation from January 2000 through March 2004, among whom personal exposures to PM_2.5_ and PAH were monitored in the second trimester of pregnancy were available for analysis. Women attending Krakow ambulatory prenatal clinics in the first two trimesters of pregnancy were eligible. Enrollment included only nonsmoking women with singleton pregnancies between 18 and 35 years of age and were free from chronic diseases, such as diabetes and hypertension. Recruited women were interviewed and given the study description and participation requirements. Subjects were given a detailed questionnaire at study entry and in the third trimester to solicit data on demographics, house characteristics, date of the last menstrual period (LMP), medical and reproductive history, occupational hazards, alcohol consumption, and smoking practices of others present in the home. After birth, maternal and child hospital records were reviewed to obtain data on complications of delivery. Weight, length, and head circumference (HC) at birth were recorded for all infants. Gestational age at birth was defined as the interval between the last day of the mother’s LMP and the date of birth. Finally we analyzed data of 455 children as preterm births (<37 weeks of gestation age) and outliers (PM_2.5_ and PAH concentrations above 95% of their distributions) were excluded from analysis.

### Dosimetry of prenatal personal exposure to PM_2.5_ and PAH compounds

During the second trimester, a member of the air monitoring staff instructed the women in the use of the personal monitor, which is lightweight, quiet and worn in a backpack. The women wore the monitor during daytime hours for two consecutive days and placed it near the bed at night. On the morning of the second day, the air monitoring staff person and interviewer visited the women’s homes to change the battery pack and administer the full questionnaire. They checked that the monitor had been running continuously and that no technical or operating failures had occurred. A staff member returned to the women’s homes on the morning of the third day to pick up the equipment. A personal environmental monitoring sampler (PEMS) measured particle mass. The PEMS is designed to achieve the particle target size of ≤2.5 μm at a flow rate of 4.0 L min with an array of 10 impactor nozzles. Flow rates were calibrated (with filters in place) before the monitoring and were checked again with the battery pack change on the second day and at the conclusion of the monitoring. Pumps operated continuously at 2 L/min over the 48 h period. To modify the sampler to achieve the 2.5-μm size cutoff at 2 L/min, five of the nozzles were blocked. Particles were collected on Teflon membrane filter (37 mm Teflo; SKC, Inc., Eighty-Four, PA, USA). The combination of low pressure drop (permitting use of a low-power sampling pump), low hygroscopicity (minimizing bound water interference in mass measurements), and low trace element background (improving analytical sensitivity) of these filters makes them highly appropriate for personal particle sampling. Dust air samples were analyzed by the Department of Environmental Health, School of Public Health, Harvard University (JD Spengler).

The air extracts were analyzed at Southwest Research Institute (SWRI) by gas chromatography/mass spectrometry for pyrene and the eight carcinogenic PAH— benzo(*a*)pyrene, benz[*a*]anthracene, chrysene/is chrysene, benzo[*b*]fluoranthene, benzo[*k*]fluoranthene, indeno[*1,2,3-c,d*]pyrene, dibenz[*a,h*]anthracene, and benzo[*g,h,i*]perylene—as described (U.S. Environmental Protection Agency 1999). The recovery of the extraction surrogate, *p*-terphenyl-d14, was consistently >60%, indicating satisfactory recovery of collected PAH. The 48 matrix spikes showed that the procedure efficiently extracted all nine PAH from the filter and polyurethane foam (PUF), with recovery means ranging from 91 to 117% and recovery standard deviations from 18 to 31%. We did not adjust air concentrations for spike recoveries. Two laboratory technicians analyzed all PAH samples using the same technique. Measurement agreement of the duplicate PAH samples was >90% over the 2-year exposure assessment period. The detection limit for each target PAH was 1.0 ± 0.2 ng/sample; 100% of the air samples were above the detection limit for all PAH except for D(*a,h*)A (73% >detection limit).

For quality control (QC), each personal monitoring result was assessed for accuracy in flow rate, time, and completeness of documentation. Samples not meeting QC criteria for adequacy were not included in the analysis. We validated 48 h personal monitoring as an indicator of longer-term, integrated exposure in two ways. In a subset of NYC cohort study (*n* = 84), indoor air was monitored over six weeks during the third trimester concurrent with the personal air monitoring over 48 h The prenatal personal air concentrations were significantly correlated with indoor levels of PAH (sum of the 8 carcinogenic PAH) (*r* = 0.58, *p* value <0.001) (Rundle et al. 2012). The PAH compounds measured were significantly intercorrelated. Spearman rank correlation varied from 0.89 to 0.99. PAH_total_ (hereafter referred to as PAH) was calculated as sum of all nine monitored PAH. Correlations between PAH_total_ and monitored PAH compounds were between 0.94 and 0.99.

### Statistical methods

The main birth outcomes were birth weight, length, and HC at birth, and associations with exposure were examined by univariate and multivariate models. Preterm births (less than 37 weeks of gestation age) and outliers (PM_2.5_ and PAH concentrations above 95% of their distributions) were excluded from analysis. We constructed several models with exposures to PM_2.5_ or PAH treated as continuous. Crude effects were estimated and subsequently adjusted to confounders. All maternal factors included in multivariate analyses were related to birth outcomes in bivariate analysis. Next, we assessed the effects of PM_2.5_ and PAH exposure on each birth outcome by multiple linear regression, controlling for potential confounders (parity, maternal education, pre-pregnancy weight, weight gain during pregnancy, sex of infant, gestational age, and season of birth). We tested season of birth for confounding because of its association with exposure and potential association with fetal growth. Season of birth was introduced in the regression models as a dummy variable, with summer defined as the reference level. Because the distribution of the air pollutants was skewed, the PM_2.5_ and PAH values were log transformed before entry into the regression models. To estimate the overall effect of PM2.5 and PAH each pollutant model was run in turn (single pollutant models) and then independent effect of each pollutant was modeled by including both PM_2.5_ and PAH in the same multivariable model (two-pollutant model). The interaction term between PM_2.5_ and PAH was statistically non-significant in all outcomes (*p* > 0.3), so it was not included in final models. As PM_2.5_ and PAH were significantly inter-correlated (*r* = 0.58, *p* < 0.0001), we tested multicollinearity between log transformed measures using Variance Inflated Factors (VIF). VIFs were equal to 1.51, which is an acceptable level for inclusion in the same model (Kutner et al. 2005).

In all multivariable models, in addition to unstandardized regression coefficient, the standardized Beta coefficients are presented, to compare the effect sizes of relationships between the two studied pollutants and birth outcomes. The Beta coefficients are interpreted as effect of a change of one standard deviation in the ln-transformed pollutant level. Additionally, ln-transformed values of PM_2.5_ and PAH were transformed into *z* scores and presented on graphs with various patterns of the association.

## Results

Table 1 presents the study sample characteristics according to the season of delivery. The sample was well-balanced through the seasons. There were very small differences between particular parameters across the season groups; however infants delivered in autumn had a somewhat lower mean birth weight compared to those born in other seasons.

**Table 1:** Study sample characteristics by season of infant’s delivery

Variables	Season of delivery				Total *N* = 455
	Spring *N* = 124	Summer *N* = 112	Autumn *N* = 117	Winter *N* = 102	
Maternal age (years)	27.4 ± 3.68	27.7 ± 3.55	27.5 ± 3.56	27.9 ± 3.36	27.6 ± 3.54
Maternal education (years)	15.6 ± 2.82	15.5 ± 2.69	15.5 ± 2.81	15.8 ± 2.71	15.6 ± 2.75
Parity	41 (33.1%)	43 (38.4%)	45 (38.5%)	37 (36.3%)	166 (36.5%)
Gestational age (weeks)	39.6 ± 1.18	39.6 ± 1.10	39.6 ± 1.16	39.5 ± 1.06	39.6 ± 1.12
Pre-pregnancy weight (kg)	57.3 ± 7.82	58.9 ± 8.76	57.3 ± 7.48	59.5 ± 10.31	58.2 ± 8.61
Weight gain during pregnancy (kg)	16.1 ± 4.73	15.2 ± 5.07	15.5 ± 4.90	15.5 ± 5.85	15.6 ± 5.12
Child’s sex (males)	67 (54.0%)	57 (50.9%)	62 (53.0%)	50 (49.0%)	236 (51.9%)
Birth weight (g)	3465 ± 437.5	3481 ± 408.4	3406 ± 433.2	3482 ± 450.2	3457 ± 432.0
Birth length (cm)	54.8 ± 2.51	55.0 ± 2.44	54.6 ± 2.71	54.9 ± 2.76	54.8 ± 2.60
HC at birth (cm)	33.9 ± 1.44	33.9 ± 1.25	34.0 ± 1.41	33.9 ± 1.53	33.93 ± 1.40
Delivery method (caesarian section)	21 (16.9%)	15 (13.4%)	21 (18.0%)	21 (20.6%)	78 (17.1%)
Exposure to environmental tobacco smoke present	24 (19.4%)	24 (21.4%)	28 (23.9%)	27 (26.4%)	103 (22.6%)

Table presents mean ± SD and *n* (%)

Personal PM_2.5_ median values (μg/m^3^) measured in pregnant women during the second trimester varied between 27.3 μg/m^3^ in the non-heating season (May–September) and 43.4 μg/m^3^ in the heating season (October–April) (Table 2). The highest PM_2.5_ values were recorded during November–December and January–February and the lowest values were reported during May–September. There was especially large seasonal variability of PAH personal exposure between the non-heating and heating periods. Median values of PAH concentrations varied between 7.6 ng/m^3^ in the non-heating season and 52.9 ng/m^3^ in the heating season. Similar to PM_2.5_, recorded values of PAH values were highest during the months November–December and January–February and were lowest during the period May–September (Figure S1). The median level of PAH measured in the heating season was sevenfold higher compared with that in the non-heating season.

**Table 2:** Personal exposure to individual PAH compounds, total PAH, and PM_2.5_ monitored prenatally (median values with quartile range)

Variables	Total sample *n* = 455	Non-heating (May–September) *n* = 196	Heating season (October–April) *n* = 259	Heating/non-heating ratio
Benzo(a)anthracene (ng/m^3^)	1.9 (0.7–6.1)	0.6 (0.5–0.9)	5.62 (3.1–10.4)	9.2
Benzo(b)fluoranthene (ng/m^3^)	3.5 (1.4–10.3)	1.3 (0.9-2.0)	9.31 (5.0-16.7)	7.2
Benzo(k)fluoranthene (ng/m^3^)	1.1 (0.5–3.2)	0.43 (0.3–0.7)	2.86 (1.6–4.9)	6.7
Benzo(g,h,i)perylene (ng/m^3^)	2.0 (0.8–5.4)	0.72 (0.5–1.1)	5.07 (2.8–8.5)	7.0
Benzo(a)pyrene (ng/m^3^)	2.2 (0.7–6.8)	0.64 (0.4–0.9)	6.27 (3.3–12.2)	8.7
Chrysene/iso-chrysene (ng/m^3^)	2.0 (0.8–5.7)	0.73 (0.5–1.1)	5.28 (2.8–8.8)	7.2
Dibenzo(a, h)anthracene (ng/m^3^)	0.4 (0.1–1.2)	0.09 (0.09–0.2)	1.10 (0.6–1.9)	12.2
Indeno(1,2,3-c,d)pyrene (ng/m^3^)	2.4 (0.9–6.5)	0.78 (0.6–1.1)	6.31 (3.3–10.3)	8.1
Pyrene (ng/m^3^)	4.0 (2.2–10.1)	2.16 (1.7–2.9)	8.92 (4.6–14.6)	4.2
total PAH (ng/m^3^)	19.0 (7.8–54.8)	7.57 (6.0–11.2)	52.85 (27.2–89.1)	7.0
PM_2.5_ (µg/m^3^)	34.0 (22.6–49.7)	27.33 (19.5–37.7)	43.38 (26.8–58.2)	1.6

**Fig. 1:**
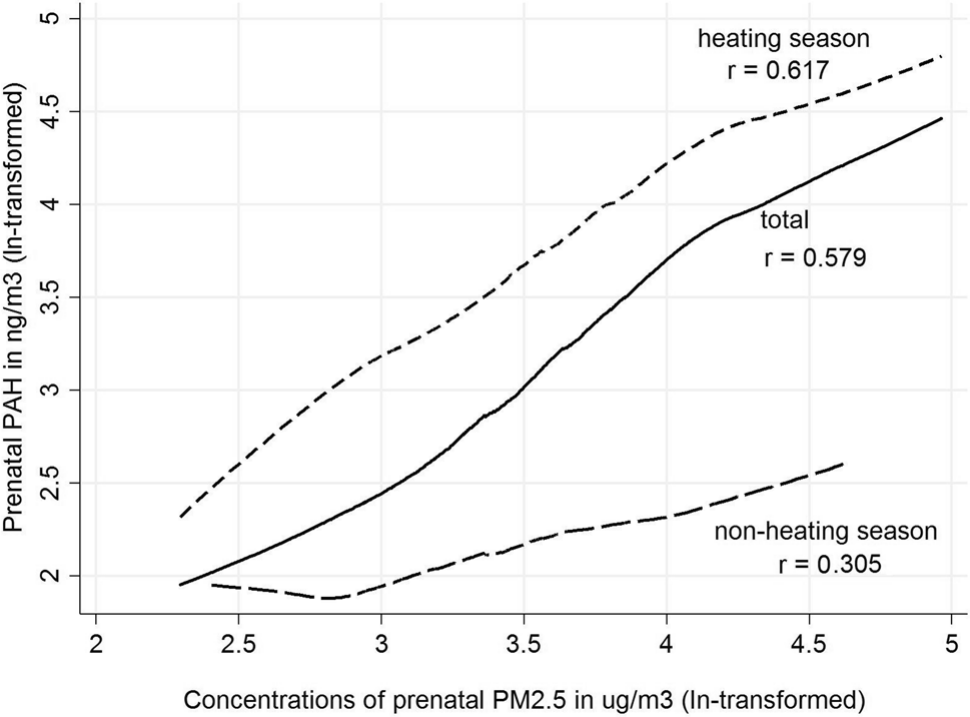
Comparison of the relationship between PM_2.5_ and PAH (ln-transformed) concentrations measured in the 2nd trimester of pregnancy, grouped by season and in the total study group

**Fig. 2:**
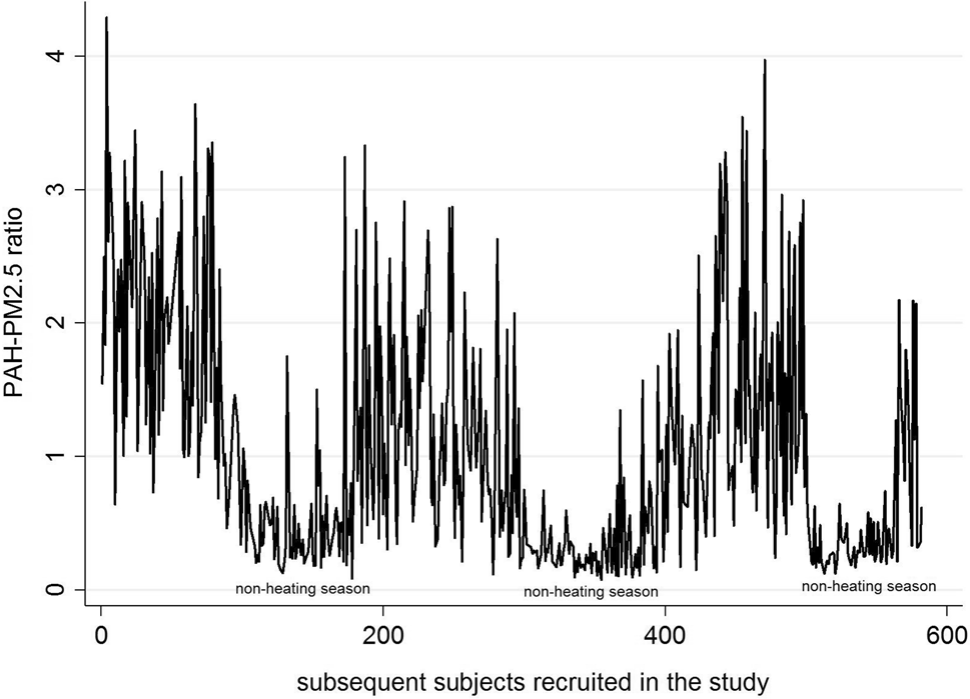
Individual variability of the PAH-PM_2.5_ ratio in study subjects over the heating and non-heating seasons

Pairwise correlation coefficients between PAH and PM_2.5_ were lower in the non-heating season (*r* = 0.305, *r* ^2^ = 9.3%) than in the heating season (*r* = 0.617, *r* ^2^ = 38.1%) (Fig. 1). To estimate inter-individual variability in prenatal exposure to PAH and PM_2.5_ due to location within the Krakow inner city area behavior and activities, we calculated the PAH: PM_2.5_ concentration ratio among individual residents in the study. The mean PAH-PM_2.5_ ratio was about five-fold higher in the heating season compared with that observed in the non-heating season (1.49 vs. 0.34). Figure 2 shows that we observed large variability of the PAH-PM_2.5_ ratios calculated for each participant in the study across the seasons, and between individual participants within each season.

Table 3 shows the results of single-pollutant and two-pollutant multivariate linear regression models to determine the impact of prenatal exposure to PM_2.5_ and PAH (ln-transformed) on birth outcomes, where the unstandardized coefficients and the standardized effect size of the exposure (Beta coefficients) were appraised. All potential confounders, except from environmental tobacco smoke, introduced in the models were statistically significant. Full models are available in supplemental materials (Tables S1-S3).

**Table 3:** Unstandardized and standardized coefficients (Beta) for prenatal PM_2.5_ (µg/m^3^, ln-transformed) and PAH exposure (ng/m^3^, ln-transformed) on birth parameters

	Prenatal PM_2.5_ (ln-transformed)				Prenatal PAH (ln-transformed)			
	Coef	*t*	*P* > *t*	*β*	Coef	*t*	*P* > *t*	*β*
Birth weight (g)								
Single-pollutant approach with PM_2.5_^a^	−78.83	−2.29	0.023	−0.10				
Single-pollutant approach with PAH^a^					−81.87	−3.71	<0.001	−0.21
Two-pollutant approach^a^	−12.71	−0.31	0.757	−0.02	−77.04	−2.89	0.004	−0.20
Birth length (cm)								
Single-pollutant approach with PM_2.5_^a^	−0.54	−2.40	0.017	−0.11				
Single-pollutant approach with PAH^a^					−0.47	−3.25	0.001	−0.20
Two-pollutant approach^a^	−0.20	−0.75	0.453	−0.04	−0.39	−2.25	0.025	−0.17
HC (cm)								
Single-pollutant approach with PM_2.5_^a^	−0.30	−2.46	0.014	−0.11				
Single-pollutant approach with PAH^a^					−0.22	−2.80	0.005	−0.17
Two-pollutant approach^a^	−0.16	−1.09	0.277	−0.06	−0.16	−1.72	0.086	−0.13

Single-pollutant and two-pollutant approach

^a^Adjusted to maternal education (years), child sex, parity, maternal pre-pregnancy weight (kg), weight gain in pregnancy(kg), gestational age (weeks), exposure to environmental tobacco smoke (yes vs. no) and birth season

### Birth weight

Both PM_2.5_ and PAH exposures were found individually to be inversely associated with birth weight. Newborns experienced a birth weight deficit of 78.8 g (unstandardized coefficients) with an increase of PM_2.5_ by one ln unit; the corresponding birth weight deficit in newborns exposed in the prenatal period to PAH amounted to 81.9 g by one ln unit. However, the standardized PAH effect size was twice that of PM_2.5_ (*β* = − 0.21 vs. *β* = − 0.10).

In the two-pollutant model the newborns exposed to PM_2.5_ showed a mean birth weight deficit of 12.7 g per increase of PM_2.5_ by one ln unit; the corresponding birth weight deficit attributed to PAH exposure in the prenatal period amounted to 77.0 g. The standardized and adjusted size effect of PAH in the two-pollutant model was highly significant and about ten times stronger (*β* = −0.20, *p* = 0.004) than that attributed to PM_2.5_ (*β* = −0.02, *p* = 0.757). The latter model results suggest that the negative effect of PM_2.5_ on birth weight in the single-pollutant model may have been mediated by the PAH component of PM_2.5_.

### Birth length

Both exposure variables in the single pollutant models were inversely and significantly associated with birth length. The newborns exposed prenatally to PM_2.5_ showed an average deficit in length of 0.5 cm for an increase of PM_2.5_ concentration by one ln unit; the corresponding deficit in birth length in infants exposed to PAH amounted to 0.5 cm. In terms of the standardized Beta coefficients, however, the deficit for PAH was about twice (*β* = −0.20, *p* = 0.001) that attributed to PM_2.5_ (*β* = −0.11, *p* = 0.017). In the two-pollutant regression model for prenatal exposure to PM_2.5_ and PAH on birth length, infants prenatally exposed to PM_2.5_ showed a mean deficit of 0.20 cm for an increase in the concentrations of PM_2.5_ by one ln unit; the corresponding deficit with PAH amounted to 0.39 cm. However, the Beta coefficient for PAH exposure in the two-pollutant model was roughly three times (*β* = −0.17, *p* = 0.025) that for PM_2.5_ (*β* = −0.04, *p* = 0.453). As in the two-pollutant approach for birth weight, the effect of PM_2.5_ on birth length appears to have been mediated by PAH.

The adjusted PAH effect size on birth weight and length estimated in the two-pollutant model appeared to be approximately the same strength (*β* = −0.20, *p* = 0.004 for birth weight vs. *β*= −0.17, *p* = 0.025 for birth length).

### Head circumference

Both exposure variables in the single pollutant models were inversely and significantly associated with HC. The newborns prenatally exposed to PM_2.5_ showed an HC deficit of 0.30 cm per PM_2.5_ concentration increase of one ln unit; the corresponding HC deficit in infants exposed to PAH in the prenatal period amounted to 0.22 cm. In terms of the adjusted Beta coefficients, the size effects of both pollutants analyzed in the single pollutant models were roughly equal.

In the two-pollutant regression models for prenatal exposure to PM_2.5_ and PAH on HC, the newborns prenatally exposed to PM_2.5_ showed an adjusted HC deficit of 0.16 cm per increase in PM_2.5_ concentration by one ln unit; the HC deficit in infants exposed to PAH in the prenatal period amounted to 0.16 cm. The adjusted size effect of PAH established in the two-pollutant model was roughly twice of that of PM_2.5_, however they both appeared to be insignificant (Table 3).

### Patterns of relationships between PM_2.5_ and PAH and birth outcomes

**Fig. 3:**
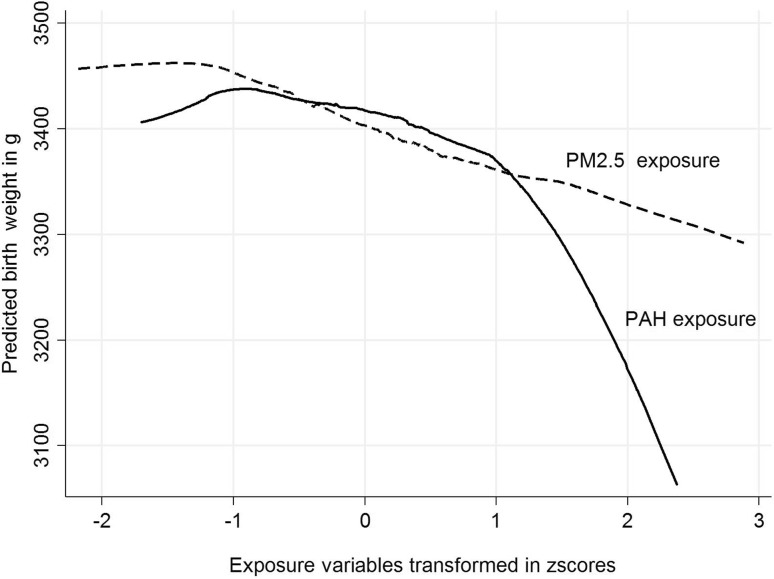
Patterns of the relationship between prenatal exposure to PM_2.5_ and PAH (concentrations transformed to *z* scores) for predicted birth weight (g)

**Fig. 4:**
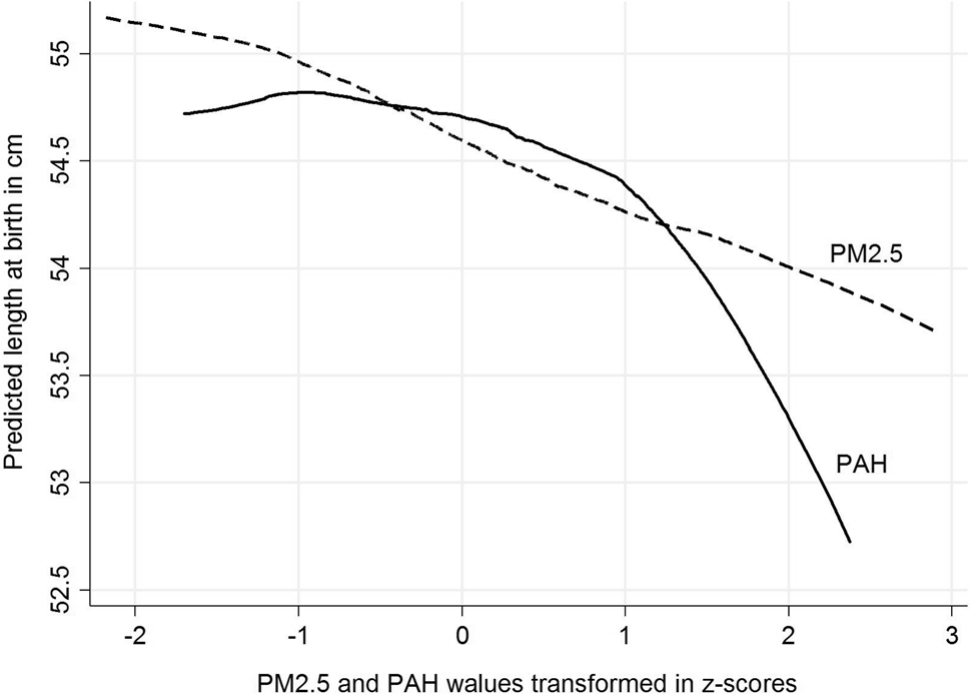
Patterns of the relationship between prenatal exposure to PM_2.5_ and PAH (concentrations transformed in *z* scores) for predicted birth length (cm)

**Fig. 5:**
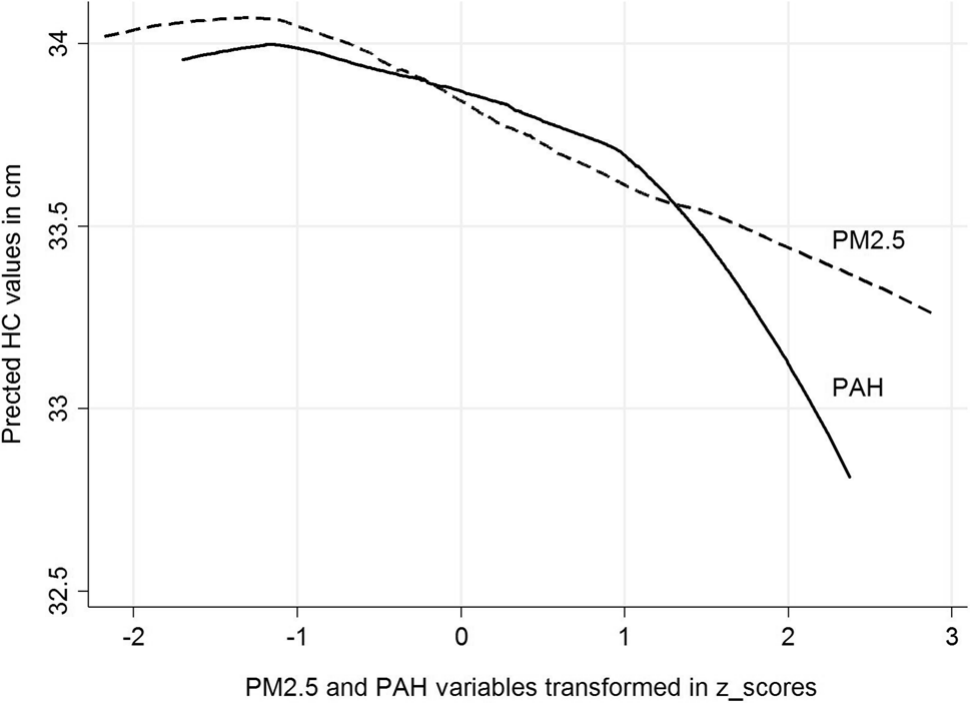
Patterns of the relationship between prenatal exposure to PM_2.5_ and PAH (concentrations transformed in *z* scores) for predicted HC at birth (cm)

Figure 3 presents the pattern of relationships between the concentrations of prenatal PM_2.5_ or PAH and birth weight, where the concentrations of prenatal exposure were transformed to *z* scores. Prenatal PM_2.5_ exposure was associated with birth weight deficit in a linear manner, over both lower and higher ranges of exposure; however, the relationship between prenatal PAH exposure and birth weight was curvilinear. The shape of the relationships between the concentrations of both prenatal exposures (transformed to *z* scores) and length and HC at birth (presented in Figs. 4, 5) is similar to that observed for birth weight.

## Discussion

This is the first birth prospective cohort study to assess the associations between prenatal personal exposure to fine particles and PAH on birth outcomes based on single- and two-pollutant approaches. As expected, the airborne personal concentrations of PM_2.5_ and PAH measured in women in the second trimester of pregnancy were correlated with each other; however the pairwise correlation between PM_2.5_ and PAH was lower in the non-heating (*r* = 0.305, *r* ^2^ = 9.3%) than in the heating period (*r* = 0.617, *r* ^2^ = 38.1%), suggesting large seasonal variation in the PM_2.5_ chemical composition and implying that fine particles in the heating season were more heavily loaded with PAH compounds compared with the non-heating period. The mean PAH-PM_2.5_ ratio was about five times higher in the heating season compared with the non-heating season. Irrespective of season, PM_2.5_ concentrations explained only 33.5% of variability of PAH. Our observations are consistent with PM chemical composition data, which varies greatly across regions and seasons, affecting toxicity (Bell et al. 2007; Dominici et al. 2006; Peng et al. 2008, 2009). The data also show large variability in the chemical composition of fine particles between individual residents within each season, which may be attributed, in part, to local emission points (burning coal for home heating) in the cold season. Krakow’s emission sources are typical of many areas in the new EU member states, especially in Central and Eastern Europe, and major developing countries like China and India (Junninen et al. 2009). Thus, results of our study may be generalized to similar metropolitan areas in Europe and Asia.

In the single-pollutant approach both pollutants were significantly associated with deficits in all birth outcomes (birth weight, length and HC) after adjustment for potential confounding factors and exclusion of preterm newborns and outliers. Importantly, the PAH effect size on birth weight and length was twice that of PM_2.5_. The effect sizes of both pollutants on HC in the single pollutant approach were also statistically significant, and were similar.

In the two-pollutant models, the negative effect of PM_2.5_ on birth weight, adjusted for PAH exposure, lost its significance, suggesting that the PAH component of PM_2.5_ fraction is more biologically relevant to birth outcomes than PM. Some residual confounding by PM_2.5_ could have resulted since fine particles measured in the study contained other compounds (other PAH compounds, metals, nitrates, sulfates etc.) not detected by the PAH measurement procedures but known to have potential negative health effects. The standardized effect size of PAH on birth weight established from the two-pollutant model was highly significant and about ten times larger (*β* = −0.20, *p* = 0.004) than that attributed to PM_2.5_ (*β* = −0.02, *p* = 0.757); and the standardized effect of PAH on birth length was two times greater than that for PM_2.5_. In contrast, PAH did not appear to have a greater effect on HC than PM_2.5_.

Comparing the patterns of associations between PM_2.5_ and PAH (*z* score transformed), while prenatal PM_2.5_ exposure was associated with birth outcomes in a linear manner, equally through lower and higher ranges of exposure, the relationship between PAH exposure and all birth outcomes was curvilinear, suggesting that both exposure factors may lead to detrimental effects on the fetal development through different biologic mechanisms.

The biologic mechanisms whereby PM_2.5_ and PAH might cause adverse pregnancy outcomes are not well established. PM_2.5_ might be a proxy measure of a complex of toxic agents present in the environment, including PAH, that could adversely affect fetal growth. Fine particles are also virtually always present in combustion processes that generate other toxic agents. Typically, the ambient fine particle fraction contains organic compounds, sulfates, metals, and soot (Spengler et al. 2001). Therefore, PM_2.5_ represents a wide spectrum of environmental health hazards implicated in intrauterine fetal growth and is rather a poor surrogate of specific etiological agent responsible for deficits in birth outcomes.

The mechanisms by which fetal PAH exposure impacts birth outcomes may include the induction of apoptosis after DNA damage, anti-estrogenic effects and binding to human aryl hydrocarbon receptor to induce P450 enzymes to receptors for placental growth factors, resulting in decreased exchange of oxygen and nutrients (Davila et al. 1996; Herbstman et al. 2012; Lutz et al. 1998; Page et al. 2002; Perera 2011).

This analysis suggests that the PAH component of PM_2.5_ may be mediating the negative effect of PM_2.5_ on birth weight and length and that the interpretation of single-pollutant studies in terms of causality may be of limited value for public health policy. Greater public health protection from air pollution could be achieved by shifting from a single pollutant approach to a multi-pollutant approach (Dominici et al. 2010). Though this approach has been explored in cardiovascular and respiratory epidemiology, few reproductive epidemiology studies have assessed this. Another challenge, and limitation of the present study, is the assessment of combined exposures and interactions between co-pollutants.

A considerable strength of the study is the Krakow birth cohort design, which included individual personal measurements of the main exposure variables among women in the second trimester of pregnancy with an established QC system (Choi et al. 2008; Rundle et al. 2012). This methodological approach provided an improved integrated pattern of the personal indoor and outdoor exposure considering the mobility of subjects within the monitoring period than the air pollution data obtained from previous ecologic or “semi-individual studies” where air pollution was estimated by a few monitors that might not represent individual exposures. This could result in exposure misclassification, which is prone to effect estimates biased toward the null.

In the present study, relevant confounders such as child sex, gestational age, parity, pre-pregnancy weight and weight gain over pregnancy, and maternal education as a proxy of socio-economic status were accounted for. Maternal health characteristics possibly linked with the infant’s health or maternal exposure to active smoking were controlled by the exclusion criteria.

## Conclusions

PM_2.5_ is considered a useful surrogate for a multi-pollutant factor in epidemiologic studies. However, interpretation of PM_2.5_ data in terms of a causal relationship with adverse birth outcomes is problematic, owing to the large temporal and spatial variability in its content of PAH and other potential hazardous compounds, even within a relatively small urban residential area. The results suggest that, in order to protect population reproductive health, public health policy should also focus on specific components such as PAH, shown here to be more strongly associated with birth weight and length than PM_2.5_.

## Electronic supplementary material

Below is the link to the electronic supplementary material.

Supplementary material 1 (JPG 34 KB)

Supplementary material 2 (DOCX 81 KB)
