# Supplementary figures and images for: Prenatal exposure to fine particles and polycyclic aromatic hydrocarbons and birth outcomes: a two-pollutant approach
Source: Int Arch Occup Environ Health. 2017 Feb 7;90(3):255–64. doi: 10.1007/s00420-016-1192-9 (PMC5360842; doi:10.1007/s00420-016-1192-9)

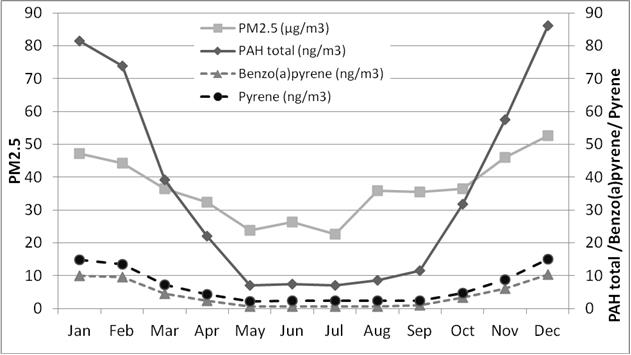

Supplement: Supplementary file 1 — Supplementary material 1 (JPG 34 KB) [file 420_2016_1192_MOESM1_ESM.jpg]
