# Supplementary material for: Prenatal exposure to fine particles and polycyclic aromatic hydrocarbons and birth outcomes: a two-pollutant approach
Source: Int Arch Occup Environ Health. 2017 Feb 7;90(3):255–64. doi: 10.1007/s00420-016-1192-9 (PMC5360842; doi:10.1007/s00420-016-1192-9)
Supplement: Supplementary file 2 — Supplementary material 2 (DOCX 81 KB) [file 420_2016_1192_MOESM2_ESM.docx]

**Supplemental Material:**

PRENATAL EXPOSURE TO FINE PARTICLES AND POLYCYCLIC AROMATIC HYDROCARBONS AND BIRTH OUTCOMES. A TWO-POLLUTANT APPROACH

Wieslaw A. Jedrychowski, Renata Majewska, Jack D. Spengler, David Camann, Emily L. Roen, Frederica P. Perera

**Table of Contents:**

| Table S1. | Pg. 3 |
| --- | --- |
| Table S2. | Pg. 4 |
| Table S3. | Pg. 5 |
| Figure S1. | Pg. 6 |

Table S1. Unstandardized and standardized coefficients (Beta) for prenatal PM_2.5_ (µg/m^3^, ln-transformed) and PAH (ng/m^3^, ln-transformed) exposure on birth weight (g). **Single-pollutant (model 1, 2) and two pollutant approach (model 3)**

|  |  |  |  |  |
| --- | --- | --- | --- | --- |
|  | Coef. | t | P>t | Beta |
| **model 1** |  |  |  |  |
| Prenatal PM_2.5_ | -78.83 | -2.29 | 0.023 | -0.1 |
| Maternal education (yrs) | 15.65 | 2.41 | 0.016 | 0.1 |
| Child’s sex (female) | -187.51 | -5.62 | <0.001 | -0.22 |
| Parity | 145.91 | 4.2 | <0.001 | 0.16 |
| Pre-pregnancy weight (kg) | 14.78 | 7.58 | <0.001 | 0.29 |
| Weight gain in pregnancy (kg) | 24.45 | 7.23 | <0.001 | 0.29 |
| Gestational age (weeks) | 137.29 | 9.25 | <0.001 | 0.36 |
| Environmental tobacco smoke exposure (yes vs. no) | -54.71 | -1.26 | 0.209 | -0.05 |
|  |  |  |  |  |
| **model 2** |  |  |  |  |
| Prenatal PAH | -81.87 | -3.71 | <0.001 | -0.21 |
| Maternal education (yrs) | 16.17 | 2.52 | 0.012 | 0.10 |
| Child’s sex (female) | -185.89 | -5.64 | <0.001 | -0.22 |
| Parity | 138.35 | 4.03 | <0.001 | 0.15 |
| Pre-pregnancy weight (kg) | 15.4 | 7.95 | <0.001 | 0.31 |
| Weight gain in pregnancy (kg) | 24.45 | 7.31 | <0.001 | 0.29 |
| Gestational age (weeks) | 139.06 | 9.46 | <0.001 | 0.36 |
| Environmental tobacco smoke exposure (yes vs. no) | -78.81 | -1.89 | 0.059 | -0.08 |
|  |  |  |  |  |
| **model 3** |  |  |  |  |
| Prenatal PM_2.5_ | -12.71 | -0.31 | 0.757 | -0.02 |
| Prenatal PAH | -77.04 | -2.89 | 0.004 | -0.20 |
| Maternal education (yrs) | 16.1 | 2.5 | 0.013 | 0.1 |
| Child’s sex (female) | -186.4 | -5.64 | <0.001 | -0.22 |
| Parity | 139.06 | 4.03 | <0.001 | 0.16 |
| Pre-pregnancy weight (kg) | 15.36 | 7.9 | <0.001 | 0.31 |
| Weight gain in pregnancy (kg) | 24.5 | 7.3 | <0.001 | 0.29 |
| Gestational age (weeks) | 138.95 | 9.43 | <0.001 | 0.36 |
| Environmental tobacco smoke exposure (yes vs. no) | -74.42 | -1.7 | 0.089 | -0.07 |

*all models adjusted additionally for birth season

Table S2. Unstandardized and standardized coefficients (Beta) for prenatal PM_2.5_ (µg/m^3^, ln-transformed) and PAH (ng/m^3^, ln-transformed) exposure on birth length (cm). **Single-pollutant (model 1, 2) and two pollutant approach (model 3)**

|  | Coef. | t | P>t | Beta |
| --- | --- | --- | --- | --- |
| **model 1** |  |  |  |  |
| Prenatal PM_2.5_ | -0.54 | -2.4 | 0.017 | -0.11 |
| Maternal education (yrs) | 0.04 | 0.97 | 0.334 | 0.04 |
| Child’s sex (female) | -0.98 | -4.5 | <0.001 | -0.19 |
| Parity | 0.50 | 2.22 | 0.027 | 0.09 |
| Pre-pregnancy weight (kg) | 0.07 | 5.38 | <0.001 | 0.23 |
| Weight gain in pregnancy (kg) | 0.11 | 4.94 | <0.001 | 0.22 |
| Gestational age (weeks) | 0.68 | 7.03 | <0.001 | 0.30 |
| Environmental tobacco smoke exposure (yes vs. no) | -0.44 | -1.53 | 0.126 | -0.07 |
|  |  |  |  |  |
| **model2** |  |  |  |  |
| Prenatal PAH | -0.47 | -3.25 | 0.001 | -0.20 |
| Maternal education (yrs) | 0.04 | 1.06 | 0.290 | 0.05 |
| Child’s sex (female) | -0.97 | -4.46 | <0.001 | -0.19 |
| Parity | 0.46 | 2.02 | 0.044 | 0.08 |
| Pre-pregnancy weight (kg) | 0.07 | 5.68 | <0.001 | 0.24 |
| Weight gain in pregnancy (kg) | 0.11 | 4.95 | <0.001 | 0.21 |
| Gestational age (weeks) | 0.69 | 7.18 | <0.001 | 0.30 |
| Environmental tobacco smoke exposure (yes vs. no) | -0.61 | -2.23 | 0.026 | -0.10 |
|  |  |  |  |  |
| **model 3** |  |  |  |  |
| Prenatal PM_2.5_ | -0.20 | -0.75 | 0.453 | -0.04 |
| Prenatal PAH | -0.39 | -2.25 | 0.025 | -0.17 |
| Maternal education (yrs) | 0.04 | 1.02 | 0.306 | 0.05 |
| Child’s sex (female) | -0.98 | -4.49 | <0.001 | -0.19 |
| Parity | 0.47 | 2.07 | 0.039 | 0.09 |
| Pre-pregnancy weight (kg) | 0.07 | 5.61 | <0.001 | 0.24 |
| Weight gain in pregnancy (kg) | 0.11 | 4.97 | <0.001 | 0.22 |
| Gestational age (weeks) | 0.69 | 7.14 | <0.001 | 0.3 |
| Environmental tobacco smoke exposure (yes vs. no) | -0.54 | -1.87 | 0.062 | -0.09 |

*all models adjusted additionally for birth season

Table S3. Unstandardized and standardized coefficients (Beta) for prenatal PM_2.5_ (µg/m^3^, ln-transformed) and PAH (ng/m^3^, ln-transformed) exposure on HC at birth (cm). **Single-pollutant (model 1, 2) and two pollutant approach (model 3)**

| Predictors | Coef. | t | P>t | Beta |
| --- | --- | --- | --- | --- |
| **model 1** |  |  |  |  |
| Prenatal PM_2.5_ | -0.3 | -2.46 | 0.014 | -0.11 |
| Maternal education (yrs) | 0.05 | 2.36 | 0.019 | 0.1 |
| Child’s sex (female) | -0.75 | -6.48 | <0.001 | -0.27 |
| Parity | 0.51 | 4.19 | <0.001 | 0.17 |
| Pre-pregnancy weight (kg) | 0.04 | 5.94 | <0.001 | 0.25 |
| Weight gain in pregnancy (kg) | 0.06 | 5.09 | <0.001 | 0.22 |
| Gestational age (weeks) | 0.26 | 5.06 | <0.001 | 0.21 |
| Environmental tobacco smoke exposure (yes vs. no) | -0.18 | -1.2 | 0.23 | -0.05 |
|  |  |  |  |  |
| **model 2** |  |  |  |  |
| Prenatal PAH | -0.22 | -2.8 | 0.005 | -0.17 |
| Maternal education (yrs) | 0.05 | 2.41 | 0.016 | 0.11 |
| Child’s sex (female) | -0.75 | -6.47 | <0.001 | -0.27 |
| Parity | 0.49 | 4.06 | <0.001 | 0.17 |
| Pre-pregnancy weight (kg) | 0.04 | 6.17 | <0.001 | 0.26 |
| Weight gain in pregnancy (kg) | 0.06 | 5.08 | <0.001 | 0.22 |
| Gestational age (weeks) | 0.27 | 5.14 | <0.001 | 0.21 |
| Environmental tobacco smoke exposure (yes vs. no) | -0.27 | -1.85 | 0.065 | -0.08 |
|  |  |  |  |  |
| **model 3** |  |  |  |  |
| Prenatal PM_2.5_ | -0.16 | -1.09 | 0.277 | -0.06 |
| Prenatal PAH | -0.16 | -1.72 | 0.086 | -0.13 |
| Maternal education (yrs) | 0.05 | 2.4 | 0.017 | 0.11 |
| Child’s sex (female) | -0.75 | -6.48 | <0.001 | -0.27 |
| Parity | 0.49 | 4.07 | <0.001 | 0.17 |
| Pre-pregnancy weight (kg) | 0.04 | 6.1 | <0.001 | 0.26 |
| Weight gain in pregnancy (kg) | 0.06 | 5.11 | <0.001 | 0.22 |
| Gestational age (weeks) | 0.27 | 5.14 | <0.001 | 0.21 |
| Environmental tobacco smoke exposure (yes vs. no) | -0.22 | -1.46 | 0.145 | -0.07 |

*all models adjusted additionally for birth season

**
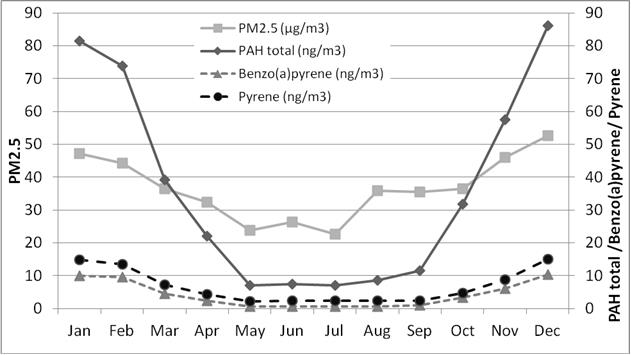
**

**Figure S1.** Seasonal variability of PM_2.5_, PAH, benzo(a)pyrene, and pyrene measured in the second trimester of pregnancy in study subjects.
